# Simplified Method of Estimating the A_300_ Micropore Content in Air-Entrained Concrete

**DOI:** 10.3390/ma16072752

**Published:** 2023-03-29

**Authors:** Jerzy Wawrzeńczyk, Henryk Kowalczyk

**Affiliations:** Faculty of Civil Engineering and Architecture, Kielce University of Technology, Al. Tysiąclecia Państwa Polskiego 7, 25-314 Kielce, Poland; zmsjw@tu.kielce.pl

**Keywords:** concrete, air-void distribution, frost resistance, air entrainment, spacing factor, A_300_ micropore content

## Abstract

It is well known that the most important parameters for predicting the frost resistance of pavement concrete are the air pore spacing factor, L, and the micropore content, A_300_. The A_300_ parameter requires complex calculations with the estimation of the air-void size in a 3D space. The procedure is based only on one-dimensional chord lengths. The air-void distribution is used only to determine the content of micropores and has no other practical application. Based on the results of the analysis, it was found that there is a simpler way to estimate the A_300_ parameter without the tedious calculations described in the EN 480-11 Standard. The presented approach is based on the existing linear correlation between the A_300_ parameter and the number of chords in 28 length classes. The developed function includes only a few coefficients (eight classes) because only chord lengths of 10–350 µm are statistically significant. This fact is important not only for the simplification of calculations but may also have consequences for the methodology of testing parameters characterizing the structure of air-entrained concrete using the 2D method. The presented function allows the estimation of A_300_ with a standard error not exceeding 0.02%, so it is useful for practical use.

## 1. Introduction

Since the 1950s, it has been known that air entrainment of concrete is the basic technological treatment that largely determines its frost resistance [1]. The method of determining the parameters of the air pore structure was included in the ASTM C 457 Standard [2]; now, the basic measurement method is based on the number and length of chords on the surface of the sample. The parameter determining the frost resistance of concrete is the spacing factor of the air bubbles, L. Two factors influence the L parameter: the specific surface area of the air pores (α) and the ratio of the volume of the cement paste to the volume of air in the concrete (P/A). The specific surface area of the pores is a simple relationship directly related to the average length of the measured chords [2].

Many concrete frost resistance tests performed in the 1990s led to the development of Standard 480, which is based primarily on ASTM C457. A new element has been introduced to the method of estimating the pore size distribution in space (3D), based on the analysis of the chord length distribution (1D) divided into 28 chord length classes. As a result, this method allows for estimating the content of micropores with a diameter of less than 300 μm (the so-called A300) [3,4]. Based on the results of research [5,6] on the use of polymer microspheres to air-entrain the concrete mix, it can be concluded that concrete can be frost-resistant even when the A_300_ micropore content is about 1%. On the other hand, an A_300_ condition above 2.0% does not guarantee the spacing of the air bubbles L < 0.20 mm. Many documents assume that concrete frost-resistance criteria concern both the requirement for spacing factor L < 0.20 mm and the content of A_300_ > 1.5% [7,8] or A_300_ > 2.0%, according to Dag Vollset et al. [9]. However, the micropore content is regarded as an auxiliary criterion of secondary importance by the ACI recommendation and the ASTM C 457 Standard. In these documents, the A_300_ criterion is not considered at all.

Air voids in concrete are characterized by a relatively large dimension range, ranging from a few micrometres to a few millimetres. The distribution of the pore dimension is not uniform; most pores (chords) are in the range of up to 200 μm, and there are far fewer above that [6,10]. In addition, it can be stated that the distribution of chords is not continuous. In some classes, there are many chords, and, in others, there are few or no chords. In the Saltykov method, the author assumed that there must be at least seven chord classes, but no more than fifteen (the table with coefficients was adapted to manual measurements). Currently, there are no such computational problems, so, theoretically, more classes can be accepted. However, the values of the transfer coefficients are very small, which, with the small number of chords in a class, should not be of significant importance [11,12,13]. The basic observation from microscopic examinations is the fact that, on the plane of the sample surface, there is an overrepresentation of large pores in relation to their number observed in the volume of concrete. Therefore, it is necessary to correct the number of chords in particular classes in the calculation. The aforementioned correction consists in converting the number of 1D chords in a given class to the number of diameters in the 3D space.

Usually, the number of chords in concrete, according to EN-480-11 [3], ranges from 400 to 1200. In the calculation of the micropore content according to the table of the EN-480-11 Standard, the ninth column (air content in each class) often contains a negative value of the pore volume, which, of course, does not make any physical sense [3]. The fact that one chord with a length of 3000 μm is equal to 300 chords of 30 μm long shows that taking into account large pores in the calculations has a significant impact on the result of the spacing factor, L. Hence the criticism made by many researchers of the method is that the spacing factor depends very much on one factor, which is the average length of the chords. It has been postulated that the calculations should take into account the actual size distribution of the chord length, and not only the average chord length [5,14,15]. Various approaches to the issue have been proposed, including [6,15,16,17,18] and others. In general, the linear traverse method measurements are carried out manually, measuring all the chords that cross air voids. It is interesting to note that the EN 480-11 Standard mentions the possibility of automating such measurements [3]. This requires a suitable contrast between the air voids and the cement paste and aggregate, which is achieved by covering the surface of the concrete with dark ink and filling the pores with white powder. Fonseca and Scherer presented a comprehensive study on the process of contrasting the surface for testing. They tested various types of white powders with different grain sizes to fill the pores [16]. In his work, Załocha presented the idea of using automatic image analysis to measure chord lengths with greater accuracy [17]. The advantage of the automatic approach is that it can provide better reproducibility of the results compared to the classic manual method. This idea was further developed [6,16], describing a 2D approach based on two-dimensional cross-sections of pores on the surface of the test sample. As a result, the exact diameter of the pores, not just the chords, could be analysed. Additionally, a qualitative analysis of each measured air void is possible because it is described by many parameters that characterise the shape, e.g., equivalent diameter, circumference, roughness, and elongation.

Hasholt [18], on the other hand, analysed the air pore structure results from four independent laboratories. In conclusion, the total surface area of air voids parameter performed as well or better than the spacing factor in determination of the frost resistance of concrete. Therefore, the total surface area parameter may be a better indicator of frost resistance.

Theoretically, the content of A_300_ could be verified, e.g., by using computed tomography (CT). At present, the resolution of the devices is too small in relation to the dimensions of the small pores, and such an estimation is unreliable [19].

This article presents an analysis of the air pore structure conducted on 292 pavement concretes (262 concretes used for the analysis and another 30 to verify the results) with different air content. Parameters A, A_300_, and α were determined by the Rosival linear traverse method, counting the length of the chords according to the EN-480-11 Standard. The aim of the analysis was to determine the relationship between the number of chords in individual classes and the content of A_300_ micropores. A statistical analysis using the linear multiple regression method made it possible to determine the correlation relationships and the significance of the influence of the selected pore ranges (classes) with the A_300_ parameter. A positive verification of the obtained regression functions would allow the proposal of a simpler, faster A_300_ calculation method without using either tedious standard calculations or estimating the air-void distributions.

## 2. Materials and Methods

**Figure 1:**
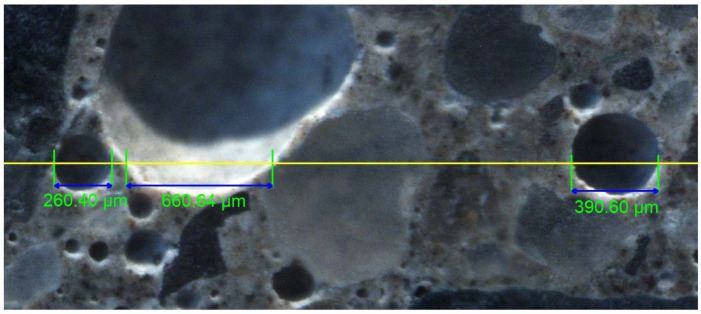
Surface of concrete with a marked traverse line.

The subject of the analysis is a dataset containing the results of measurements allowing us to characterise the air pore structure in air-entrained concretes. Microscopic examinations were carried out according to the EN-480-11 Standard using the linear traverse method. For each concrete, two 100 × 150 × 40 mm samples were prepared. The test surfaces were ground first with fine corundum powders with thicknesses of #120, #400, #600, and #800. Then, the entire surface was polished. The final surface should have a matte sheen with no visible irregularities between the cement paste and the aggregate. The natural surfaces were examined using a stereoscopic microscope (NIKON SMZ1500) with a mounted camera (NIKON DS-Fi1) and side-lighting to distinguish the air voids (according to the lighting procedure described in ASTM 457). The images were captured while the stage-mounted specimen was moving along the traverse line. A total of 28 frames (1280 × 960 pixels with a resolution of 1.13 μm/px) were merged to form a large colour image, about 80 mm in length. The NIS-Elements software was used to further analyse the output image. On the images of each sample, 15 traverse lines of 1200 mm were drawn, so that the total length of the traverses on the two samples was 2400 mm. Along each traverse, the length of the chords that crossed the air voids was measured manually. The prepared surface with applied traverse line is shown in Figure 1.

The software records individual chord lengths and allows the exporting of the recorded data to an MS Excel spreadsheet. The output data file is a table containing all the measured chords, the total length of the chords, the total length of the traverse, and the volume of the cement paste (P). All the calculations were performed according to EN 480-11 using a prepared VBA macro in MS Excel software. The result was the essential characteristics of the air-void system: total air content, A, spacing factor, $\bar{L}$, and air-void specific surface area, α. The EN 480-11 Standard additionally includes a procedure that can be used to determine the air-void distribution in a 3D space. The Standard describes a table to divide chords into 28 length classes (0–4000 μm) and calculate the air content of each class. As a result, the values of the content of micropores, A_300_, were read directly from the distribution table as the volume attributed to all the air voids with diameters ranging from 0 to 300 μm.

The collected dataset includes parameters determined for 262 concretes, such as air content, A, micropore content, A_300_, specific surface area, α, and a number of chords, N, (chord length range of 0–4000 μm).

**Figure 2:**
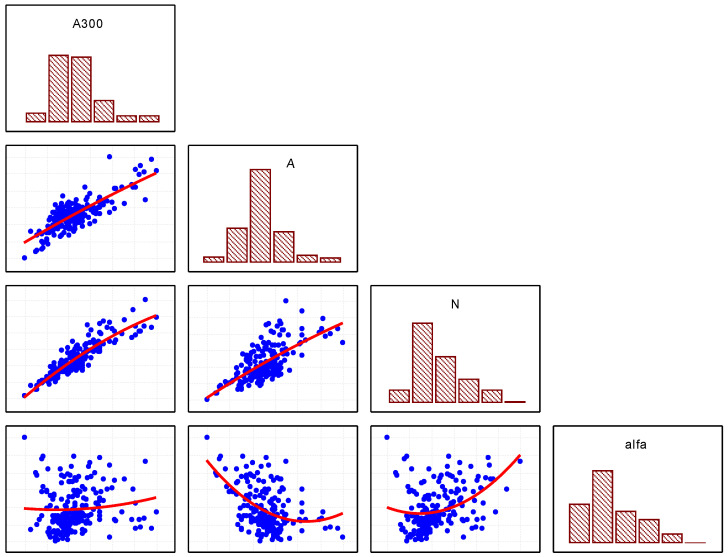
Matrix diagram showing the relationships between factors A, A_300_, α, and N (the column variables are used as X coordinates and the row variables represent the Y coordinates).

Mathematical analysis of the dataset allows the air pore arrangement to be described using parameters: A, A_300_, α, and L. The general characteristics of the set are presented in the form of a matrix diagram (Figure 2) and in Table 1.

As the above data indicate, the subject of the analysis is a very large set of concretes characterised by strongly varying air pore structure parameters.

## 3. Results

The EN-480-11 Standard provides a detailed description of the calculation procedure to determine the pore distribution in a 3D space based on the number and length of chords. On this basis, the A_300_ micropore content is determined. The calculated value of A_300_ is only a certain estimate of the actual micropore content of the space.

By observing the pore dimensions on the surface of the polished section, it is found that there is some overrepresentation of larger pores in relation to the actual pore diameter distribution in the 3D space. It is therefore necessary to correct the measured chord lengths to obtain the distribution of the diameters in the space. The starting point for the calculation is the number of chords per class.

Relationship analyses were performed using the classical linear multiple regression method. The basis of the classical regression analysis method is that both the dependent variable and the independent variables are random. The assumption that the random components have a normal distribution allows for statistical inference, i.e., the construction of confidence intervals and the use of statistical tests based on Student’s t-distribution. However, not all independent variables play a significant role in the regression function. The variables for which the regression coefficients are not significantly different from zero should be removed from the model, and a model with fewer explanatory variables should be built. The final linear multiple regression model in which all the regression coefficients are significant will only be obtained in the second or third stage. At each stage, the regression coefficients of a given model are estimated, their statistical significance is checked, and variables with regression coefficients not significantly different from zero are removed. The detection of autocorrelation (testing the hypothesis of the existence of autocorrelation) uses statistical tests such as the von Neumann or Durbin–Watson test.

To obtain a reliable regression function, it is recommended that the number of observations is 10 to 20 times the number of variables in the model. Therefore, a comprehensive dataset was prepared that included information on 262 concretes.

The multiple regression analysis assumed a linear function in the following form:

(1) $$Y=\frac{2500}{T_{tot}}\;*\;(a_{0}+a_{1}\;*\;c+a_{2}\;*\;c2+\ldots +a_{28}\;*\;c28+e)$$

where:

a_0_, a_1_, …, a_28_—regression coefficients;

c1, c2, …, c28—the number of chords recorded in a given class;

Y—dependent variable (A_300_ micropore content);

T_tot_—the length of the measuring line (total for 2 samples).

The length of the measuring line, T_tot_, (Table 1) ranged from 2438 mm to 2643 mm (mean 2522 mm), which significantly affected the number of chords recorded. Therefore, it was necessary to introduce a conversion factor in the equation, bringing the line length to 2500 mm.

**Figure 3:**
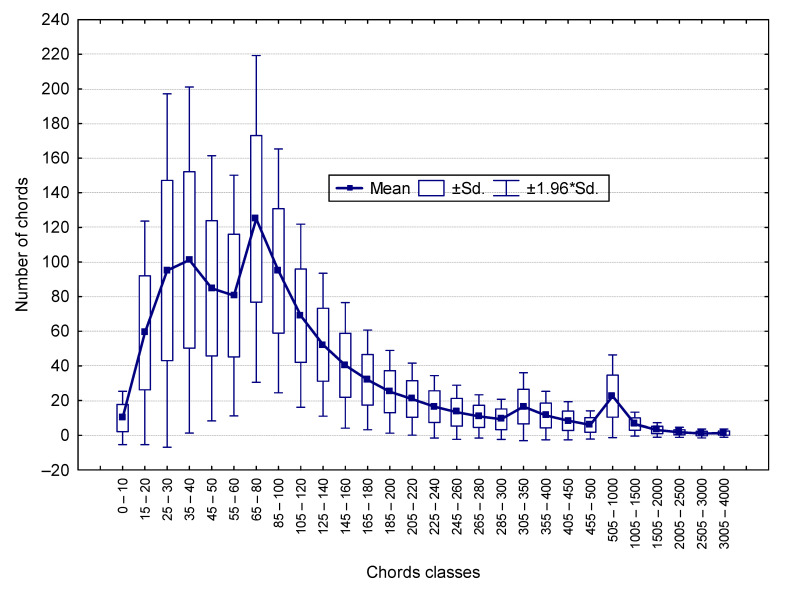
Characteristics of the distribution of the number of chords in each class.

As can be seen in Figure 3, the distribution of the number of chords in each class varies greatly. The largest number of chords is in the range of up to 200 μm. The larger the dimensions, the smaller the number of chords.

The purpose of the study was to determine the relationship between the number of chords in each class and the micropore content with a diameter of less than 300 μm (A_300_). The analysis was carried out in three stages, eliminating variables (chord classes) with a non-significant impact on A_300_ and grouping the others into classes with larger dimension ranges. This led to the development of a linear function with only eight regression coefficients. A number of analyses were carried out to verify the goodness of fit of the function with the measurement data, verifying the possible autocorrelation of the independent variables and the normality of the distribution of the residuals as key conditions for the quality of the relationship obtained. All the statistical calculations were performed using Statistica software.

**Stage 1** : Analysis of the dataset including all 28 chord classes

The dataset for the calculations is shown schematically in Table 2.

The multiple regression analysis yielded the following results:

R = 0.99999, R^2^ = 0.9999, Adj.R^2^ = 0.9999;

df = 28,233, F = 441694E3, *p* = 0.0000;

Standard error of estimation: 0.00013, Std. error: 0.000037;

t(233) = −2.233, *p* = 0.0265;

where:

R—multiple correlation coefficient;

R^2^—coefficient of determination;

Adj.R^2^—corrected goodness of fit;

Df—degrees of freedom; 

p—significance value.

**Stage 2: Analysis of 18 chord classes**

In this stage of the study, linear regression function calculations were performed considering only 18 chord classes in the range of 15–350 μm. The following multiple regression results were obtained:

R = 0.9999, R^2^ = 0.9999, Adj. R^2^ = 0.9999;

df = 18,243, F = 690648E3, *p* = 0.0000 Standard error of estimation: 0.00013;

Std. error: 0.0000318, t(243) = −2.732 *p* = 0.0068.

BETA regression coefficients in Table 3 refer to the regression function calculated for standardised input variables and B for raw (non-standardised) variables. The values of the Beta coefficients allow a comparison of the relative contribution that each of the independent variables makes to the prediction of the dependent variable. Based on the table of coefficients of the regression function for the 28 pore classes, it was found that only the coefficients corresponding to the chord classes of 15–350 μm have a significant impact on the outcome variable of A_300_. Small pores with chords of 0–10 μm have a small volume and number, which has little effect on the volume gain of the A_300_ micropores. The pores with larger dimensions, corresponding to chord lengths greater than 350 μm, do not have a significant impact on the A_300_ value. According to the stereology assumptions, larger pores can also generate chords in smaller classes, but the number of such pores is too small to affect the A_300_. The results obtained provide the basis for reducing the number of necessary chord classes from 28 to 19, and thus the number of corresponding coefficients in the regression (Equation (1)).

**Figure 4:**
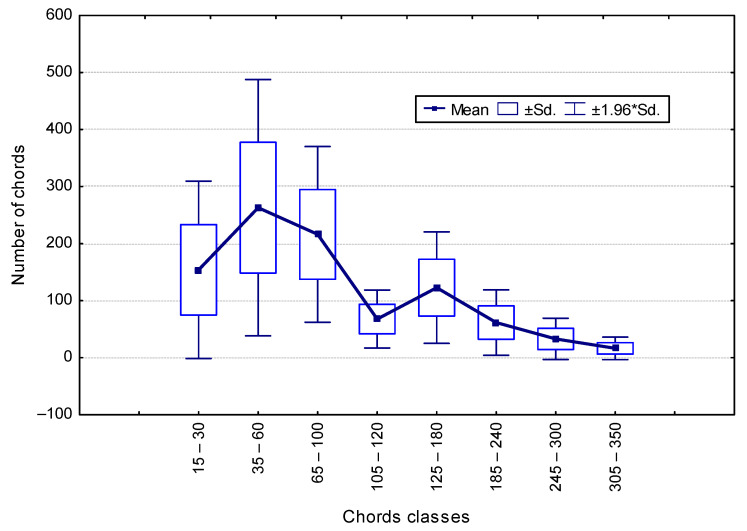
Distribution of chords for eight classes.

The comparison of the Beta coefficients shows that, for some classes, the values are similar. This was the basis for merging the classes, resulting in a smaller number of them with a larger scope. Only eight classes were separated, for which the results of the calculations are given below. Figure 4 shows a graph of the chord distribution for the eight classes.

**Stage 3: Analysis of eight chord classes**

Again, linear regression function calculations were performed, considering only the eight chord classes in the range of 15–350 μm. The following multiple regression results were obtained:

R = 0.9999, R^2^ = 0.99982, Adj. R^2^ = 0.99981;

F(8253) = 1714E2, *p* < 0.0000 Std. estimation error: 0.0125;

Std. error: 0.0028621 t(253) = −0.952 *p* = 0.3422.

The calculations show that all the regression coefficients are statistically significant (except for the absolute term) as shown in Table 4. It is interesting to note that the BETA coefficient for class no. 8 (305–350 μm), as in the previous calculation steps, has a negative value. It is clear that these chords are too large to be included in the A_300_. The more large pores, the greater the number of chords associated with these pores in the smaller classes. This fact must be taken into account to not overestimate the value of the A_300_.

**Figure 5:**
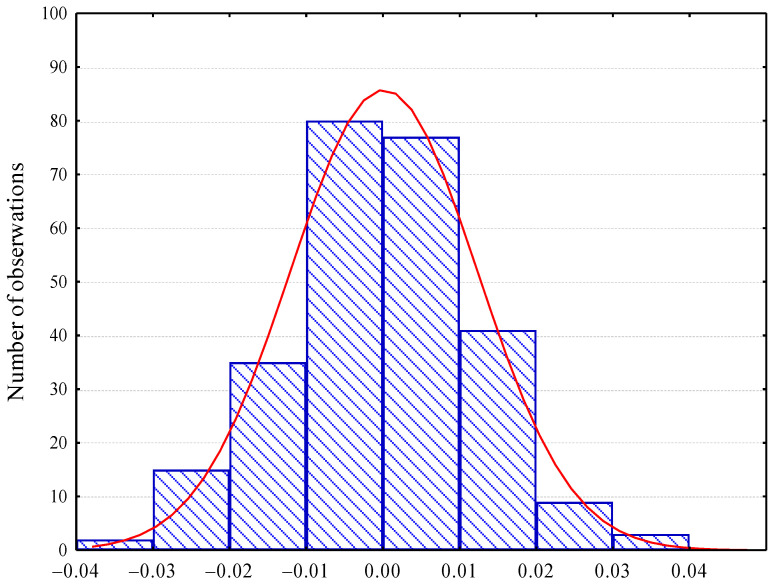
Distribution of residuals.

Figure 5 shows a comparison of the distribution of the residuals.

**Figure 6:**
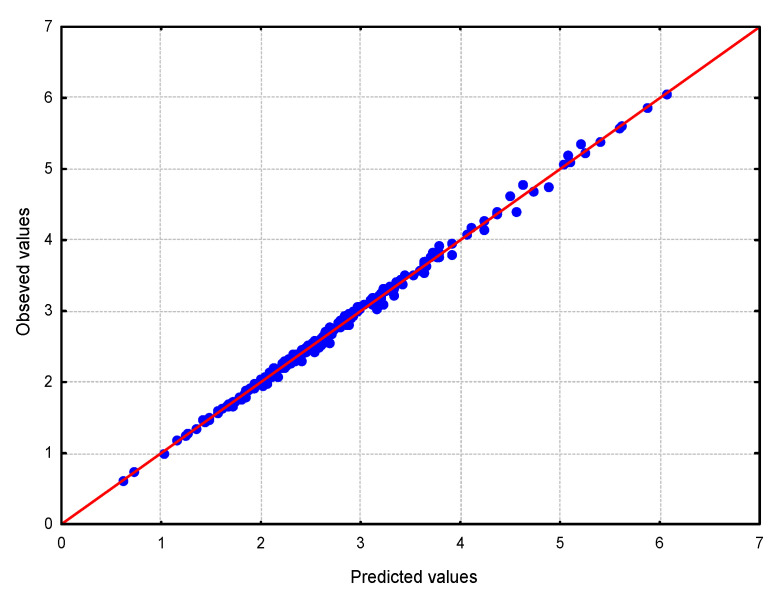
Graph of predicted vs. observed values.

The comparison between the observed values and those calculated from the regression function is shown in Figure 6. This is confirmed by the very good agreement of the calculation results with the empirical data.

The results of the analysis carried out indicate that the eight independent variables adopted allow for precisely predicting the pore content with diameters below 300 μm (R^2^ ≈ 1.0). The estimated model meets the assumptions of the least squares method. The analysis of the residuals of the analysed model confirms its validity. The value of the Durbin–Watson test statistic (DW = 1.912) allows us to conclude that there is no autocorrelation of the residuals in the resulting model.

Finally, the linear regression function for the eight separated chord length classes is shown in Table 5.

**Figure 7:**
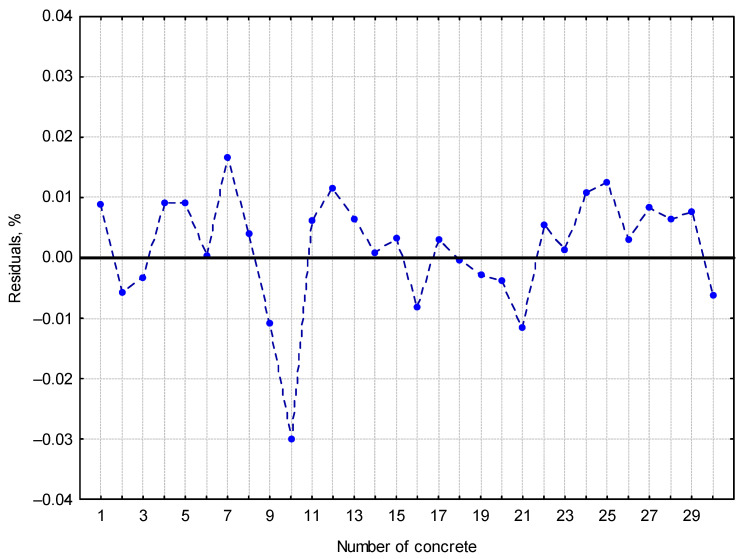
Graph of calculated residuals for 30 concretes.

In addition, an attempt was made to verify the function obtained for the data from 30 concretes that were not taken for the above calculations. The maximum difference between the calculation results and the data calculated according to EN-480-11 was 0.03%, as shown in Figure 7.

## 4. Conclusions

The purpose of the research was to develop a simple functional relationship that would allow the A_300_ micropore content to be estimated with good accuracy as an alternative to the rather complicated method contained in the EN-480-11 Standard. It was assumed that the A_300_ value could be estimated without the need to determine the distribution of pores with diameters up to 4000 μm. It should be emphasised that, apart from the A_300_ value, the pore size distribution is of no practical significance.

This article presents an analysis of an extensive dataset that contains information on 262 concretes characterised by very different air pore structures. The chord length distributions (28 classes, according to EN-480-11) are not continuous, with most chords in the range up to about 200 μm and far fewer chords of larger sizes.

The classical linear multiple regression method was used, assuming that both the independent variables (number of chords per class) and the dependent variable (A_300_) are random variables, the relationships are linear and without autocorrelation, and the residuals have a normal distribution. The tests confirmed the validity of these assumptions.

Based on the analysis results presented, it can be concluded that:

− chords with lengths of 0–10 μm and greater than 355 μm have no statistically significant effect on the A_300_ value;
− only eight classes with a chord size range of 15–350 μm are statistically significant;
− the final linear multiple regression function with eight independent variables allows us to precisely predict the A_300_ micropore content;
− the standard error of the calculation is approximately 0.02%, which means that the error of the A_300_ estimate does not exceed 0.1% of the volume of concrete and is therefore sufficient for practical purposes.

The analysis and conclusions presented here relate to measurements made using the linear analysis method (1D). It would be interesting to see how the above conclusions could be transferred to/used for the 2D method, which has recently been increasingly researched.

## Figures and Tables

**Table 1:** Characteristics.

	P %	T_tot_ mm	N	A %	A_300_ %	α mm^2^/mm^3^	L mm
mean	28.07	2522.56	1016	5.66	2.73	29.37	0.17
median	28.50	2537.74	937	5.59	2.55	27.19	0.17
Sd	1.94	47.74	313	1.65	0.92	7.49	0.04
Min	21.00	2438.62	251	0.95	0.61	15.80	0.08
Max	35.50	2643.62	2221	11.83	6.04	55.80	0.33
25%_cases	27.00	2479.42	819	4.74	2.14	24.33	0.14
75%_cases	29.15	2551.08	1194	6.42	3.09	34.20	0.19

Symbols: P—the volume of cement paste in concrete, T_tot_—total length of measuring lines for two samples, L—spacing factor.

**Table 2:** Schematic of the table adopted for multiple regression calculations.

Concrete Number	Number of Chords in Class No/Sizes					Content of Micropores A_300_, %
	1	2	…	27	28	
	0–10	15–20	…	3005–3500	3505–4000	
1	14	94	…	0	2	2.39
2	18	63	…	4	2	0.99
3	6	76	…	1	1	3.63
…	…	…	…	…	…	…
261	2	66	…	0	0	2.91
262	6	58	…	0	0	2.18

**Table 3:** Coefficients of the regression function with the assessment of their significance considering 18 chord classes.

#Class	BETA	St. Err.	B	St. Err.	t(243)	Statistical Sign.
**Absolute**			**−0.0001**	**0.0000**	**−2.7**	**0.0068**
15–20	0.0215	0.0000	0.0006	0.0000	1044.0	0.0000
25–30	0.0502	0.0000	0.0009	0.0000	1536.0	0.0000
35–40	0.0704	0.0000	0.0013	0.0000	2048.9	0.0000
45–50	0.0700	0.0000	0.0017	0.0000	2484.3	0.0000
55–60	0.0788	0.0000	0.0021	0.0000	2915.9	0.0000
65–80	0.1379	0.0000	0.0027	0.0000	4498.1	0.0000
85–100	0.1343	0.0000	0.0035	0.0000	4544.0	0.0000
105–120	0.1230	0.0000	0.0043	0.0000	5012.8	0.0000
125–140	0.1164	0.0000	0.0051	0.0000	4969.8	0.0000
145–160	0.1151	0.0000	0.0058	0.0000	5254.5	0.0000
165–180	0.1051	0.0000	0.0067	0.0000	4585.7	0.0000
185–200	0.0992	0.0000	0.0075	0.0000	4803.6	0.0000
205–220	0.0952	0.0000	0.0083	0.0000	4477.1	0.0000
225–240	0.0919	0.0000	0.0091	0.0000	4924.1	0.0000
245–260	0.0851	0.0000	0.0099	0.0000	4331.9	0.0000
265–280	0.0753	0.0000	0.0107	0.0000	3968.6	0.0000
285–300	0.0731	0.0000	0.0113	0.0000	4185.6	0.0000
305–350	−0.2430	0.0000	−0.0219	0.0000	−12,019.7	0.0000

**Table 4:** Coefficients of the regression function with the assessment of their significance considering only 8 chord classes.

No.	#Class	BETA	St. Err.	B	St. Err.	t(253)	Stat. Sign.
	Absolute t.			−0.0027	0.0029	−1.0	0.3422
1	15–30	0.0606	0.0019	0.0007	0.0000	31.4	0.0000
2	35–60	0.2102	0.0032	0.0017	0.0000	65.7	0.0000
3	65–100	0.2680	0.0031	0.0031	0.0000	85.8	0.0000
4	105–120	0.1237	0.0023	0.0043	0.0001	53.7	0.0000
5	125–180	0.3188	0.0028	0.0058	0.0001	113.7	0.0000
6	185–240	0.2688	0.0028	0.0083	0.0001	94.3	0.0000
7	245–300	0.2156	0.0024	0.0106	0.0001	90.1	0.0000
8	305–350	**−0.2413**	0.0018	−0.0218	0.0002	−130.8	0.0000

**Table 5:** Regression coefficient table for eight classes.

Class No.	1	2	3	4	5	6	7	8
Class size	15–30	35–60	65–100	105–120	125–180	185–240	245–300	305–350
Regression coefficient	0.0007	0.0017	0.0031	0.00043	0.0058	0.0083	0.0106	−0.0218

## Data Availability

Data sharing not applicable.
